# Combined Effect of Nitrofurantoin and Plant Surfactant on Bacteria Phospholipid Membrane

**DOI:** 10.3390/molecules25112527

**Published:** 2020-05-28

**Authors:** Monika Rojewska, Wojciech Smułek, Krystyna Prochaska, Ewa Kaczorek

**Affiliations:** Institute of Chemical Technology and Engineering, Poznan University of Technology, 60-695 Poznan, Poland; monika.rojewska@put.poznan.pl (M.R.); Krystyna.Prochaska@put.poznan.pl (K.P.); ewa.kaczorek@put.poznan.pl (E.K.)

**Keywords:** antibiotics, bacteria, membrane permeability, nitrofurantoin, phospholipids membrane, POPE, saponins, surfactants

## Abstract

Due to the increasing use of antibiotics, measures are being taken to improve their removal from the natural environment. The support of biodegradation with natural surfactants that increase the bioavailability of impurities for microorganisms that degrade them, raises questions about their effect on bacterial cells. In this paper we present analysis of the interaction of nitrofurantoin (NFT) and saponins from the *Saponaria officinalis* on the environmental bacteria membrane and the model phospholipid membrane mimicking it. A wide perspective of the process is provided with the Langmuir monolayer technique and membrane permeability test with bacteria. The obtained results showed that above critical micelle concentration (CMC), saponin molecules are incorporated into the POPE monolayer, but the NFT impact was ambiguous. What is more, differences in membrane permeability between the cells exposed to NFT in comparison to that of the non-exposed cells were observed above 1.0 CMC for *Achromobacter* sp. KW1 or above 0.5 CMC for *Pseudomonas* sp. MChB. In both cases, NFT presence lowered the membrane permeability. Moreover, the Congo red adhesion to the cell membrane also decreased in the presence of a high concentration of surfactants and NFT. The results suggest that saponins are incorporated into the bacteria membrane, but their sugar hydrophilic part remains outside, which modifies the adsorption properties of the cell surface as well as the membrane permeability.

## 1. Introduction

Excessive use of antibiotics and the lack of their control when released into the environment have contributed to deterioration of the ecosystem. Many antibiotics can get into the natural environment due to the fact that modern water treatment plants have not been designed to completely remove these substances. In consequence, antibiotics can enter the environment and accumulate in ecosystems. The presence of antibiotics in the environment can be harmful to many useful microbial communities as a consequence of their bacteriocidic and bacteriostatic actions. This problem also refers to nitrofuran derivatives, which are administered to humans and animals. Their representative derivative is nitrofurantoin (NFT), which is a broad spectrum antibiotic used to treat bladder infections of humans as well as in veterinary medicine [1,2]. NFT is only partially metabolized by the mammal organisms, while the majority of the compound is excreted unchanged and goes to the sewage and to the environment. For this reason, new solutions that would allow removal of the pharmaceutical product from the ecosystem are searched for. One of the promising methods is the biodegradation of the antibiotic in the natural environment using bacteria and saponins. Pacholak et al. [3] have shown that some bacterial strains use nitrofurantoin as a source of carbon and energy. Nitrofurantoin removal by selected microbial cultures ranged from 50% to 90% in 28 days, depending on the bacterial strain. On the other hand, this biodegradation process is quite long and relatively low effective. However, surfactants are expected to support the process to obtain its better efficiency. The presence of a surfactant promotes, among others, an increase in the permeability of biological membranes. To avoid additional contamination of the environment by artificial surfactants, the natural ones, like saponins, would be especially useful [4]. Saponins found application in enhancing biodegradation of persistent organic pollutants like halogenated phenols [5] or polycyclic aromatic hydrocarbons (PAH’s) [6]. Hence, it can be expected that saponins’ would promote the increase in bioavailability of the antibiotic for bacteria and as a result accelerate the biodegradation process [7,8,9]. However, in order to reasonably control the surfactant-enhanced biodegradation process, it is necessary to thoroughly understand the mechanism of nitrofurantoin interaction with the bacterial surface membrane in the presence of saponins at the molecular level. Currently, a lot of research has been made on the potential bioactivity of saponins focused only on the overall cellular response to many stimuli. However, these studies cannot provide clear molecular information on the mechanism of action of saponins on the membrane. The physiological mode of the cells adjustment in response to the external stimuli such as temperature or mechanical force is modification of the lipid composition of their membrane to maintain functional integrity [10]. From this point of view, the Langmuir monolayers technique is an alternative to experiments using living cell because it allows investigation of analogues of biological membranes. It is possible because a biological membrane can be considered as composed of two weakly coupled monolayers. Lipid monolayers formed at the air-water interface are a versatile membrane model for studying the interaction and physicochemical properties of monolayer components. The simplicity of creating model lipid systems allows investigation of the interaction of real biological membranes with bioactive substances such as saponins. Both the composition and molecular density of Langmuir monolayers can be easily controlled. Moreover, these model lipid systems can be easily characterized using a number of surface characterization techniques (surface pressure and surface potential, surface rheology, Brewster microscopy and fluorescence, neutron and X-ray scattering, ellipsometry or reflection spectroscopy (UV and IR) [11].

The Langmuir monolayer technique is also suitable to investigate the interaction between saponin and biological membrane [11,12]. This technique allows the creation of a monolayer imitating biomembrane by spreading organic compounds (e.g., lipids, phospholipids and glycolipids) on the aqueous subphase. In effect, amphiphilic lipids form an insoluble monolayer called the Langmuir film, where the molecules are oriented with their hydrophobic tail towards the gas phase and the hydrophilic head group anchored in the aqueous subphase. The formation of model membrane involves dissolving lipids in an organic solvent (e.g., chloroform) and then this solution is deposited onto the subphase. While the solvent evaporates, the lipid molecules spread to form a Langmuir film at the air/water interface. The measuring principle is based on the fact that the monolayer is compressed continuously by Teflon barriers during the experiment and the change in surface pressure vs. the monomolecular layer area at this time are recorded. The surface pressure is recorded by a Wilhelmy plate connected to a pressure sensor. The Wilhelmy plate is immersed into the subphase and the forces acting on the plate include gravity, surface tension and upward forces. The monolayer compressed to the surface pressure of 30 mN/m represents the natural biological membrane conditions [13,14,15]. Moreover, lipids play a very important role in maintaining the stiffness of the membrane and determine its permeability to other substances.

Hence, in this study we are concerned with finding out the ways in which saponins, NFT and their mixtures interact with the phospholipids monolayer. With these ideas in mind, we studied these interactions on a model of cellular membrane in the form of Langmuir monolayer consisting of selected lipid 2-oleoyl-1-palmitoyl-sn-glycero-3-phosphoethanolamine (POPE). The lipid part of the phospholipid represents the major fatty acids found in cell membranes structures of environmental bacteria. Saponins were extracted from *Saponaria officinalis* roots. When trying to link molecular structure with interfacial behavior it should be kept in mind that the extracts can differ in the saponins content and composition. In addition, impurities (residual plant substances) may be present and affect interfacial properties. Saponins are surface-active substances so we expect that they interact with the model membrane, but we wanted to determine in detail their interaction mechanism and establish the influence of natural surfactants concentration on the membrane stability in the nitrofurantoin environment. This knowledge seems to be crucial for the possible use of saponins as substances supporting the process of nitrofurantoin biodegradation.

## 2. Results

### 2.1. POPE Membrane Model Tests

The amphiphilic character of saponins allows them to aggregate in aqueous solutions and interact with membrane components. With this in mind we study how the change in concentration of saponins influence their interaction with the model membrane and consequently disturb the membrane fluidity. In our experiments, after formation of a phospholipid monolayer at the buffer subphase (pH = 7.4) we exchanged the subphase to deliver saponins or/and NTF to the investigated system. The subphase flow was slow enough (13 mL/min) to neither disturb the structure of the monolayer nor cause its destruction in the process of replacement of the bulk phase with a new one. The relaxation experiment consisted of keeping the surface pressure (π) constant, and recording the area (A) as a function of time.

First, POPE was spread on the buffer subphase (pH = 7.4) to form a monolayer. After 15 min, the film was compressed to the surface pressure of 30 mN/m, and after compression the surface per molecule was estimated as A_0_. When introducing *Saponaria* extract into the subphase, a change in surface value per molecule was observed in the monolayer (A(t)). If this value (A(t)) is greater than the A_0_ (A(t)/A_0_ > 1), an increase in the area per molecule caused by the embedded saponin molecule in the phospholipid monolayer is indicated. Otherwise, if A(t)/A_0_ < 1, there is a surface area loss in the monolayer, so it can be assumed that the phospholipid molecules desorb from the monolayer and dissolve in the subphase. The A/A_0_ parameter was therefore a measure of the monolayer stability.

**Figure 1:**
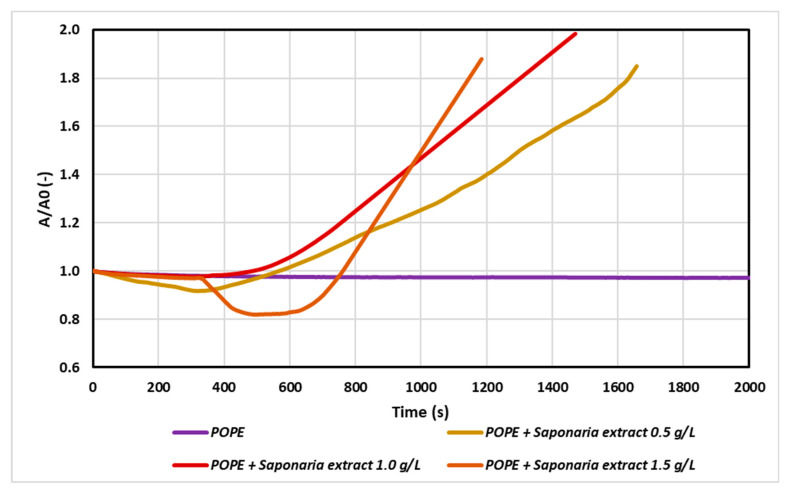
Relative area-time curves for the POPE monolayer in the control sample (with buffer only) and with saponins injected into the subphase in concentrations: 0.5 g/L; 1.0 g/L and 1.5 g/L (i.e., 0.5 of critical micellar concentration–CMC; 1.0 CMC and 1.5 CMC, respectively). The plot shows the normalized area per molecule (A/A_0_) as a function of time, where A_0_ = A (t = 0).

Two effects can be observed as a result of the interaction of the membrane with the molecules introduced in the subphase. If the particles build into the model monolayer then the surface area between the Teflon barriers will increase to maintain the set surface pressure (30 mN/m). Otherwise, we can observe a reduction of the surface area between the Teflon barriers as a consequence of desorption of molecules from the monolayer. In this way, the relaxation/penetration experiment provided important information about surface properties and stability of model membrane in the presence of saponins and NTF as a result of their mutual interactions. At first, pure lipid was spread at the water–air interface to obtain the initial surface pressure of approximately 30 mN/m. Then 150 mL of the saponins solution in various concentrations were injected into the water subphase, beneath the lipid film, to reach a final concentration of 0.5 g/L (0.5 CMC - critical micellar concentration), 1 g/L (1.0 CMC) and 1.5 g/L (1.5 CMC), respectively. In aqueous solutions at low surfactant concentrations, surfactant molecules appeared in the form of monomers. However, above a certain critical concentration called critical micellar concentration (CMC), surfactant molecules can exist as both monomers and micellar associates. On the basis of the surface tension isotherm, the CMC value of analyzed *Saponaria* extract was estimated about 1 g/L. In our experiments we chose such concentrations of saponins to permit investigation of the effect of their aggregation process on the adsorption. To avoid aggregation of saponins in the subphase, a solution of 0.5 g/L was prepared, corresponding to a saponins concentration below the CMC. The results are presented in Figure 1. The saponins penetrate from the subphase into the condensed lipid monolayer and interrupt its structure, because the A/A_0_ value increased in time.

According to the results, the injection of saponins changed drastically the monolayer relaxation behavior as the saponins penetrated from the subphase into the condensed lipid monolayers. The higher the saponins concentration in the subphase, the faster the incorporation of saponins into the monolayer was observed. When the plant extract was used at 0.5 CMC, the increase in the initial surface area value (A_0_) was doubled after about 1700 s, whereas for the test with *Saponaria* extract at 1.5 g/L (i.e., 1.5 CMC), the same effect was reached after about 1200 s.

In the next stage of our study, the effect of nitrofurantoin (NFT) concentration on the POPE monolayer was analyzed. The addition of NFT to the subphase slightly influenced the relaxation of POPE monolayers, even if the amount of antibiotic was increased significantly. At higher NFT concentrations a decrease in the A/A_0_ value over time was observed.

**Figure 2:**
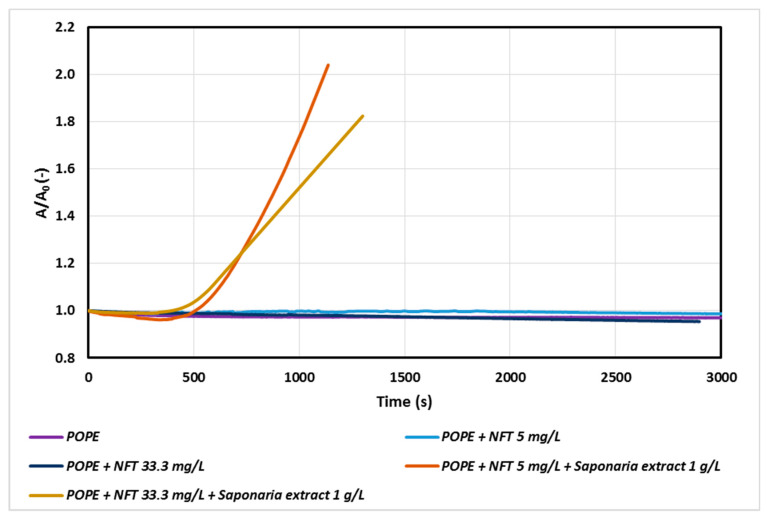
Relative area-time curves for POPE monolayer with NFT or with NFT and extract concentrations 1.0 g/L saponins injected into the subphase.

A more visible impact of NFT concentration was observed only for the systems containing saponins in the subphase (Figure 2). In the system with NFT concentration sixfold higher than that used in the previous experiment, a decrease in the value of A/A_0_ was observed after about 1000 s. Thus, a high concentration of the antibiotic in the model phospholipid membrane environment is not conducive to the process of penetration of saponins through the monolayer, especially during prolonged exposure of the membrane to these compounds. It can be assumed that there are interactions between saponins and nitrofurantoin, which begin to compete with the interactions of saponins with a phospholipid monolayer and as a result decelerate penetration process of saponins by POPE.

**Figure 3:**
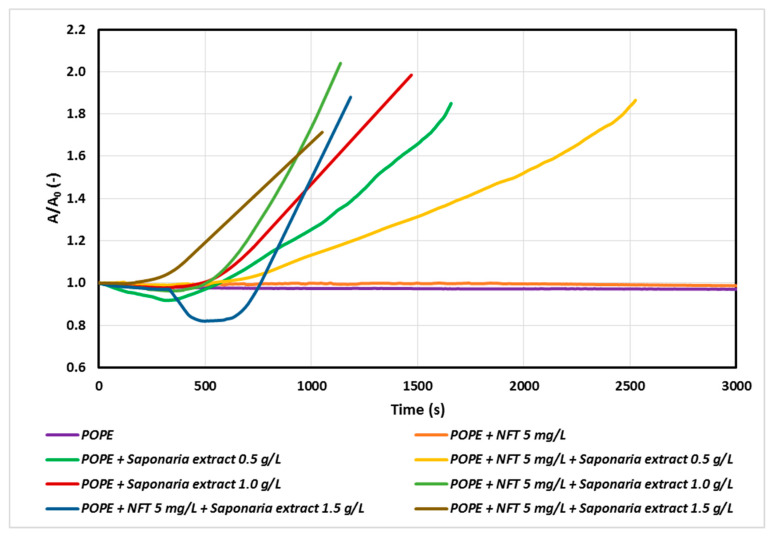
Relative area-time curves for the POPE monolayer with 5 mg/L of NFT and saponins-rich extract injected into the subphase.

At the next stage of the study, we studied the systems of a constant concentration of nitrofurantoin (5 mg/L) and the concentrations of saponins corresponding to 0.5, 1.0 and 1.5 CMC in the subphase. The results are presented in Figure 3.

The faster penetration of natural surfactant molecules into the monolayer with increasing saponins concentration was observed for both the samples with *Saponaria* extract as well as those with combined NFT and *Saponaria* extract injected. On the other hand, the character of the relaxation curves recorded for the systems with or without addition of NFT in the subphase was different from that of the above curves. However, the impact of the NFT addition on the saponins containing system is ambiguous. At the concentration of the plant surfactant corresponding to 0.5 CMC the surfactants incorporation into the phospholipid monolayer was found to decrease after 500 s. For the same system, the increase in the surface area A in the relation to the initial surface area A_0_ reached about 30% after 1500 s, whereas for the system without the addition of NFT the observed increase in area was larger and equal to about 70%. The opposite effect was obtained in the experiments with NFT and the *Saponaria* extract, in which the concentration of saponins was higher (1.0 CMC or 1.5 CMC). In this case, the addition of NFT to the systems containing saponins made the surfactant molecules to faster penetrate the phospholipid monolayer, especially at the beginning of the relaxation process.

### 2.2. Bacteria Membrane Permeability

**Figure 4:**
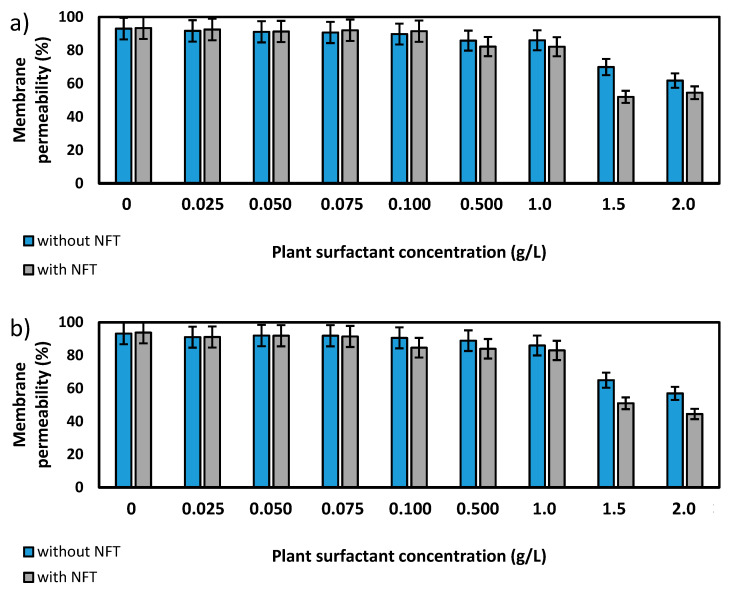

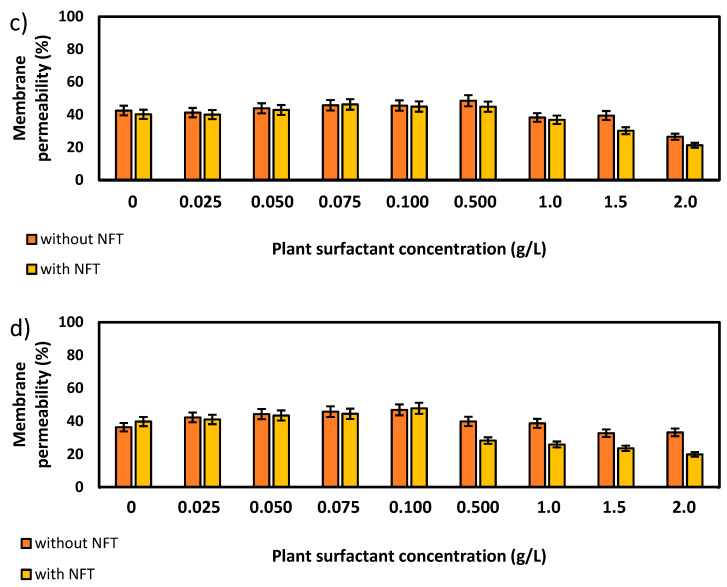
Total membrane permeability of *Pseudomonas* sp. MChB strain (**a**,**b**) and *Achromobacter* sp. KW1 (**c**,**d**); as measured after 15 min (**a**,**c**) and 120 min (**b**,**d**); NFT-nitrofurantoin.

Further experiments focused on the measurements of membrane permeability in biological systems using a crystal violet assay. The two strains, *Pseudomonas* sp. MChB and *Achromobacter* sp. KW1 of high content of fatty acids present also in POPE, were chosen for the tests. The results are presented in Figure 4. For the *Pseudomonas* sp. MChB control samples, i.e., those non-exposed to NFT or plant extract, the permeability was relatively high and exceeded 90%. Moreover, no statistically important changes in the cell membrane permeability were observed up to the critical micelle concentration equal to 1. The same observations were made after 15 min (Figure 4a) and 120 min (Figure 4b). At the plant surfactant concentrations above 1 CMC the permeability decreased significantly, which was even more noticeable after 120 min than after 15 min. After 120 min and at 1.5 CMC, the cells membrane permeability was equal to 65%. What is worth mentioning there were no differences between the cells exposed to NFT and those non-exposed to NFT below CMC. When the extract concentration exceeded CMC, the addition of NFT led to a slight decrease in the membrane permeability.

The results obtained for *Achromobacter* sp. KW1 were relatively similar, however some important differences should be indicated. The strain cells were characterized by lower membrane permeability than those of *Pseudomonas* sp. MChB, which in control samples reached ca. 40% (Figure 4c). With increasing *Saponaria* extract concentration the permeability rose up to 50% at 0.1 CMC after 15 min and at 0.5 CMC after 120 min. What is more, the further increase in the plant surfactant concentration led to a decrease in the cell membrane permeability, like it was observed for *Pseudomonas* sp. MChB cells. Moreover, at the *Saponaria* extract concentration higher than 0.1 CMC, the *Achromobacter* sp. KW1 cells exposed to NFT showed lower permeability than the cells in the samples with surfactants only.

### 2.3. Adhesion on Cell Surface

**Figure 5:**
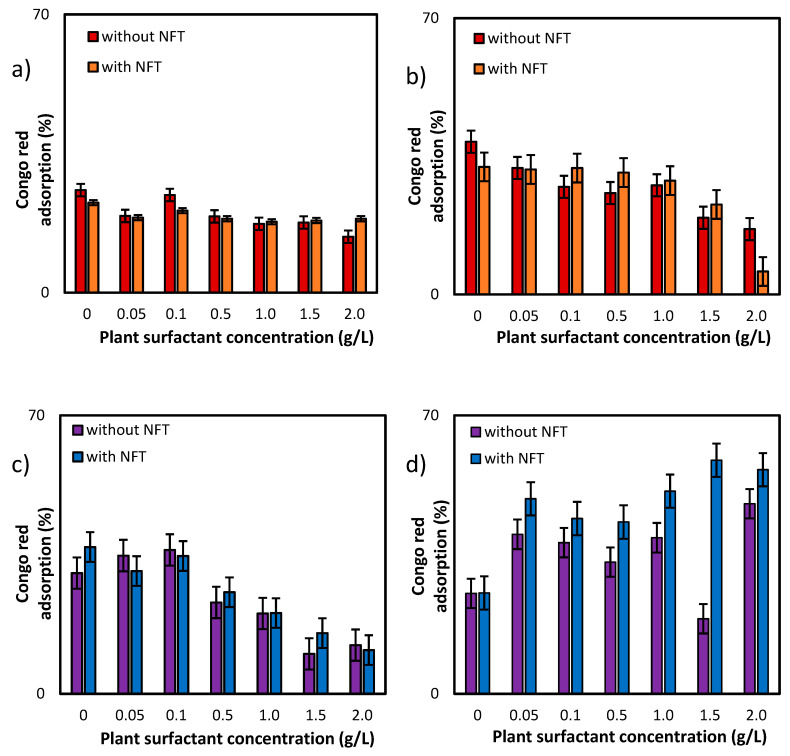
Congo red adsorption on the cell surface of *Pseudomonas* sp. MChB strain (**a**,**b**) and *Achromobacter* sp. KW1 (**c**,**d**); the measurements after 15 min (**a**,**c**) and 120 min (**b**,**d**).

Additional information about the effects of both, plant extract and NFT, was provided by the studies of adsorption of Congo red dye on the surface bacterial cells (Figure 5). For the first of the tested strains, *Pseudomonas* sp. MChB, the dye adsorption on the cells surface was at a level of 25% (after 15 min) and reached nearly 40% after 120 min. When the plant surfactant concentration increased, the adsorption slightly decreased, which was more visible when measurements were conducted after 120 min (Figure 5b). At 2 CMC the Congo red adsorption dropped down to 17%. Moreover, the additional presence of NFT limited the adsorption to 6%. For *Achromobacter* sp. KW1 the changes in dye adsorption were clearly visible already after 15 min. For the cells exposed to the surfactant only, the decrease from 30% (in control sample) to 12% at 2 CMC was observed. However, the impact of NFT exposure on the cell surface is ambiguous for both strains. Nevertheless, it could be concluded that the plant extract was more influential than NFT and the differences between the samples exposed and non-exposed to NFT (but at the same surfactant concentration) did not exceed 5 percent points. Only for *Pseudomonas* sp. MChB cells at the plant extract concentration of 2 CMC after 120 min a significant difference between the samples without and with NFT was noted.

## 3. Discussion

The biodegradation process supported by saponins is perceived as a promising solution for the removal of many toxic substances from the environment, including antibiotics. However, literature provides little information on the biodegradation of nitrofuran derivatives by environmental bacteria and on changes in the structure and metabolism of bacterial cells caused by contact with this group of compounds and the extract of saponins. Modifications at the bacterial cell membrane level are also of key importance for determination of the ability of individual strains to degrade nitrofuran derivatives in the presence of saponins. There are many literature reports [16,17] on the interaction of saponins with model cell membranes, however, not in the presence of antibiotics. Korchowiec et al. [18] have shown that saponins interact with model membranes and change the physical state of membranes by perturbing the lipid acyl chain orientation. Moreover, the chemical structure of the natural surfactant containing sugar groups as substituents, weakens their binding to the oppositely charged ions and causes the dissolution of phospholipid membranes in comparison with their chemical counterparts [19]. The important consequence is the surfactants tendency to insert into the membranes rather than dissolving them. There are suggestions [20] that saponin monomers insert into the outer leaflet and induce a rapid increase in the area difference between the two leaflets, which leads to a positive membrane curvature and formation of specific domains whose size increases over time. This process is accelerated by the presence of sugar chains, which help develop membrane defects and gradually permeabilize the membrane. However, as far as saponins solutions above CMC are concerned, the saponin micelles can locally increase concentrations of saponins and may be more conducive to the permeabilization process [4]. This is consistent with our results, as we observed an increase in surface area per molecule in the monolayer over time in the presence of saponins extract, regardless of the concentration of surfactant solution. However, the question is what is the effect of the nitrofurantoin presence in the solution on the interactions of the monolayer with saponins.

On the basis of the obtained data, NFT slightly interacts with the phospholipid monolayer leading to a reduction of the area per molecule over time. A similar effect has been reported in the study of Machado and Casei [1], who compared the stability curves of phospholipid DPPS with or without NFT. According to these authors, the process of relaxation in the presence of NFT causes a decrease in the monolayer surface pressures. Consequently, it induces a more compressible state of the monolayer. Moreover, it has been shown that the interaction between phospholipid and NFT can lead to the formation of interfacial aggregates.

However, what is important is that the presence of NFT had a significant impact on the penetration process of the monolayer through the plant surfactant molecules. However, the impact of the NFT addition on the plant extract containing system is ambiguous. In the case of premicellar saponins solution (0.5 CMC), the addition of NFT slowed down the incorporation of surfactant molecules into the phospholipid monolayer. It can be assumed that this effect is caused by the competitive interactions between saponins and NFT, which simultaneously limit the adsorption process of surfactants components on the monolayer.

The opposite effect was found for the micellar solutions of saponins. Above the CMC, the surfactant micelles may also interact with NFT but in a different way. This implicates that the micelles solubilize the antibiotic and enclose it inside them. The entrapment of the NFT inside the micelles may limit the competitive interactions that can be found in the premicellar solution. However, on the other hand, micelles could play a role as transporter of NFT towards the monolayer and release this compound during its diffusion process. Probably, released NFT can also interact with POPE, which leads to the formation of domains with greater area per molecule in comparison with those obtained in the experiments with *Saponaria* extract only. This effect is especially well visible for the saponins solution of 1.0 CMC. Machado and Casei [1] have shown that NFT can be incorporated in the phospholipids monolayer and form large aggregates. However, it should also be emphasized that the incorporation process may vary depending on the chemical structure of the polar head of each phospholipid. Therefore, for some phospholipids, despite the formation of large aggregates, which would probably disrupt the monolayer, formation of a more stable and ordered monolayer was observed.

Although the permeability of the bacterial cell membrane in the presence of various surfactants has already been analyzed, relatively few studies have been devoted to the effects of saponins, especially those present in the extract of *Saponaria officinalis*. Smułek et al. [21] have observed that the extract caused an increase in inner membrane permeability of *Pseudomonas putida* DA1 and *Achromobacter* sp. SA1 strains. However, Kaczorek et al. [5] have reported that *Quillaja saponaria* bark saponins and *Sapindus mukorossi* fruits saponins caused a significant reduction in the inner membrane permeability of *Raoultella planticola* WS2 and *Pseudomonas* sp. OS2 cells membranes.

**Figure 6:**
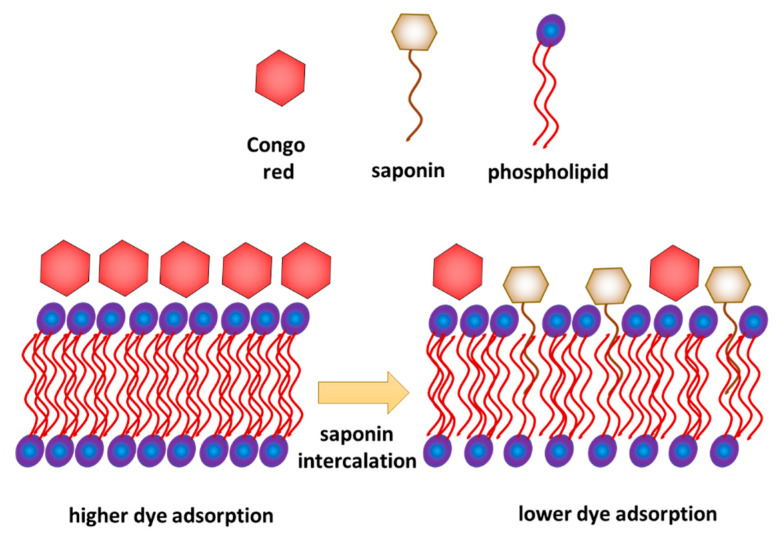
Intercalation of *Saponaria officinalis* saponins into bacterial phospholipid membrane and its influence on Congo red adsorption—proposition of the mechanism.

Combined interpretation of the results obtained from the model and biological experiments allows proposing a possible mechanism of interaction of saponins with a phospholipid membrane. On the one hand, the results of model membrane relaxation measurements indicate the saponin penetration of the membrane. On the other hand, the biological test shows a decrease in crystal violet permeation in the membrane and decrease in Congo red adsorption. It can be explained if we assume that saponins were inserted into the membrane but only with their hydrophobic part, while the hydrophilic part of saponins remained on the membrane surface (Figure 6), which limits the access of Congo red to the membrane. This interpretation is consistent with the analysis of Otzen [19], who concluded that permeation of the bacteria membrane by saponins occurs gradually and intercalation of the sugar part of the molecule happens relatively slower than the fast intercalation of the hydrophobic hydrocarbon chain.

It is worth mentioning that for both bacterial strains above the specified concentration of the plant extract, in the presence of nitrofurantoin (NFT) the membrane permeability was higher than in the samples not containing this pharmaceutical. In the case of the *Achromobacter* strain (though not so visible for the *Pseudomonas* strain) it is further observed that this difference was more significant after prolonged exposure of the cells to plant extract and NFT (Figure 4). It can be assumed that a stronger intercalation of saponins molecules into the cell membrane (at their higher concentrations) results in a change in the hydrophobicity of the cell surface and, as a result, an increased NFT absorption (contrary to Congo red adsorption—Figure 5). The presence of both hydrophilic saponins groups as well as adsorbed NFT molecules on the cell surface presumably limits cell access to crystal violet particles, whose penetration is analyzed in the used membrane permeability test. At lower concentrations of saponins, their intercalation and, as a result, the modification of the cell surface is not strong enough to affect NFT adsorption, although it is sufficient to change the permeability of the cell membrane to the crystal violet molecules.

The influence of NFT on membrane permeability is a separate issue. While in the model system with POPE the NFT impact is clear, though ambiguous, in the biological system the impact of NFT is practically imperceptible. However, it can be assumed that the model system measurements are more sensitive and the membrane reaction to NFT contact is more noticeable. In the biological system, the cell response is more strongly dominated by activity of plant surfactants.

## 4. Materials and Methods

### 4.1. Chemicals

In the presented studies, the film-forming substance was 2-oleoyl-1-palmitoyl-sn-glycero-3-phosphoethanolamine (POPE, 99%; Sigma-Aldrich, Poznan, Poland). Chloroform of high-purity Uvasol (Merck, Warszawa, Poland) was used to prepare the spreading solutions. Any other chemicals were purchased from Sigma-Aldrich, Poland. For all water solutions, ultrapure water (18 MΩ·cm, 71.98 ± 0.01 mN/m) was used. Plant extract from *Saponaria officinalis* roots (Flos, Mokrsko, Polska) was prepared in the Soxhlet apparatus (Chemland, Stargard, Poland) with methanol as a solvent according to the method described by Smułek et al. [21], and its CMC was evaluated at 1 g/L. For experiments phosphate buffer was prepared (composition in g/L: Na_2_HPO_4_∙2H_2_O 7.0, KH_2_PO_4_ 2.8, NaCl 0.5 and NH_4_Cl 1.0).

### 4.2. Model Membrane Experiments

**Figure 7:**
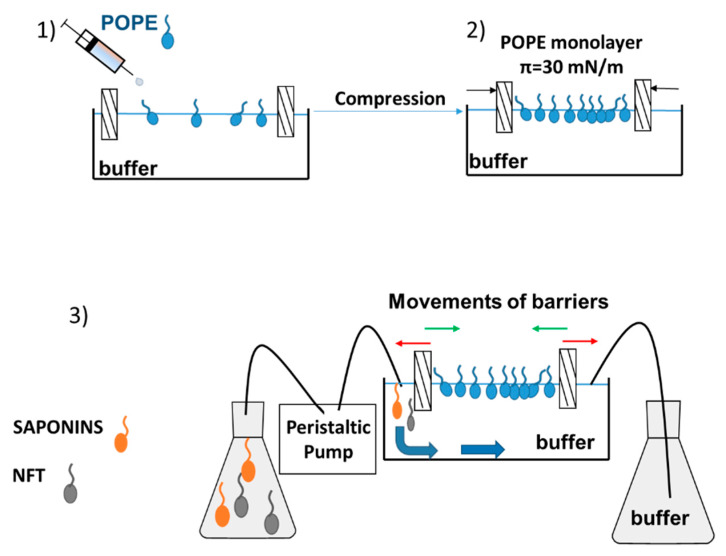
Relaxation measurement using the Langmuir monolayer technique with a dosing pump.

After formation of the phospholipid monolayer, the subphase (buffer) could be replaced with a new solution (buffer with saponins, buffer with NFT or buffer with saponins and NFT) by using a peristaltic pump (MINIPULS 3, Gilson, Middleton, WI, USA). A detailed description of the measurement has been provided in our previous paper [22]. The inlet and outlet hoses of pump were mounted to the Langmuir trough on the opposite sides, therefore the surface area of Langmuir through was constant and equal to 225 cm^2^ (Figure 7).

The following procedure was applied in the measurements:

1. Purification of the subphase (by closing the barriers and sucking the contaminations from the buffer surface) and spreading the phospholipids dissolved in a volatile solvent (chloroform),
2. Compression of the monolayer to π = 30 mN/m and waiting 15–20 min for stabilization of the film and evaporation of chloroform; in the meantime, the new subphase should be prepared and sucked into the pump tubing,
3. Buffer subphase replacement with a new one containing the additional substance/ substances, at a flow rate equal to 13 mL/min,
4. After the exchange of subphase and setting the pressure of the monolayer between the barriers at 30 mN/m the *Saponaria* extract molecules only or with NFT were observed to get adsorbed at the interface.

The relaxation experiments were performed for the pure POPE monolayer and mixed systems: POPE-saponins and POPE-saponins-NFT. The POPE film was initially compressed to a desired surface pressure 30 mN/m and then the surface area values were recorded over time. To study the POPE-saponins-NFT interactions, the POPE monolayer was compressed to a desired surface pressure and after that the saponins or saponins + NFT solution was pumped underneath the film to the subphase. The observed changes in molecular area were presented as A/A_0_, which is the ratio of the actual molecular area in time t to the molecular area at the moment of injection at a constant surface pressure. As a result of the conducted experiments, the relaxation isotherms of monolayer were obtained. The temperature of the process (25 °C) was kept constant and controlled during measurements by a Julabo F-12 circulator (Cole-Parmer, Wertheim-Mondfel, Germany).

### 4.3. Bacteria Experiments

Two environmental bacterial strains were used in the experiment: *Achromobacter* sp. KW1 (NCBI GenBank accession No. KP096516) and *Pseudomonas* sp. MChB (NCBI GenBank accession No. KU563540). The fatty acids profiles of both strains were characterized by the FAME method [23]. The dominant fatty acids were: palmitic acid (both 30%), palmitoleic acid (both 25%) and vaccenic acid (23% and 19% for *Achromobacter* sp. KW1 and *Pseudomonas* sp. MChB, respectively).

The bacteria were cultivated for 72 h with shaking at 120 rpm (KS 4000 ic control, IKA Werke GmbH, Staufen, Germany) in sterilized nutrient broth. Then, after centrifuging at 4000× *g* for 10 min (3–18K, Sigma Laborzentrifugen GmbH, Osterode am Harz, Germany) the biomass was washed twice with buffer and finally it was resuspended in the buffer to reach the final bacteria concentration of 1 × 10^9^ cfu/mL.

Total membrane permeability was tested according to [24] by colorimetric measurements of the uptake of crystal violet solution by microbial cells (CV assay). The cell surface hydrophobicity was analyzed by measuring the adsorption of Congo red dye on the surface of microbial cells (CR assay) [25]. Both measurements were conducted after 15 min and 120 min of cell exposure to NFT or/and plant extract at known concentrations.

### 4.4. Statistical Analysis

Unless otherwise noted, all experiments were performed in triplicate, then the mean value and standard deviation for the results series were calculated.

All calculations and graphs were prepared using Microsoft Excel 2013 software.

## 5. Conclusions

Micellar solutions of saponins were found to show strong interactions with the model POPE monolayer as well as the bacterial membranes. When present in concentrations above CMC, saponin molecules incorporate into the POPE monolayer, which could result in a decrease in membrane permeability. Moreover, the adhesion of the indicator, Congo red, to the membrane also decreased at high concentrations of surfactants and in the presence of NFT. On the basis of the obtained results, it was assumed that domains of the saponins could be formed in the POPE monolayer. Such domains embedded in the monolayer structure could change the fluidity of the membrane. Moreover, incorporation of saponins and NFT into the bacterial membrane could lead to a change in the membrane wettability and in consequence change its adhesion properties. In this paper we proposed the most possible mechanism of saponins and NFT interactions with the bacterial membrane.

It is noteworthy that for two different bacterial strains, but of relative similar lipids profile, changes in permeability or adhesion greatly differed, although the same concentrations of saponins and NFT were used. It indicates that other membrane components, found alongside the dominant phospholipids, significantly influence its interactions with plant surfactant or/and antibiotic. For this reason, the next stage of the study will concern the interactions between individual components of bacterial membranes with saponins and NFT. Nevertheless, it can be assumed that the saponins incorporate into the bacteria membrane with the lipid part, but their sugar hydrophilic heads remain outside the membrane, which modifies adsorption properties of the cell surface.
